# The development of mechanically formed stable nanobubbles intended for sonoporation-mediated gene transfection

**DOI:** 10.1080/10717544.2016.1250139

**Published:** 2017-02-06

**Authors:** Rodi Abdalkader, Shigeru Kawakami, Johan Unga, Yuriko Higuchi, Ryo Suzuki, Kazuo Maruyama, Fumiyoshi Yamashita, Mitsuru Hashida

**Affiliations:** 1 Department of Drug Delivery Researches, Graduate School of Pharmaceutical Sciences, Kyoto University, Kyoto, Japan,; 2 Department of Pharmaceutical Informatics, Graduate School of Biomedical Sciences, Nagasaki University, Nagasaki, Japan,; 3 Department of Drug Delivery System, Faculty of Pharma-Sciences, Teikyo University, Tokyo, Japan, and; 4 Kyoto University Institute for Integrated Cell-Material Science (iCeMS), Kyoto, Japan

**Keywords:** Nano-sized bubbles, mechanical agitation, stability, ultrasound, gene transfection

## Abstract

In this study, stable nano-sized bubbles (nanobubbles [NBs]) were produced using the mechanical agitation method in the presence of perfluorocarbon gases. NBs made with perfluoropropane had a smaller size (around 400 nm) compared to that of those made with perfluorobutane or nitrogen gas. The lipid concentration in NBs affected both their initial size and post-formulation stability. NBs formed with a final lipid concentration of 0.5 mg/ml tended to be more stable, having a uniform size distribution for 24 h at room temperature and 50 h at 4 °C. *In vitro* gene expression revealed that NBs/pDNA in combination with ultrasound (US) irradiation had significantly higher transfection efficacy in colon C26 cells. Moreover, for *in vivo* gene transfection in mice left limb muscles, there was notable local transfection activity by NBs/pDNA when combined with US irradiation. In addition, the aged NBs kept at room temperature or 4 °C were still functional at enhancing gene transfection in mice. We succeeded in preparing stable NBs for efficient *in vivo* gene transfection, using the mechanical agitation method.

## Introduction

Enhancing the delivery of nucleic acids is a major task in the field of drug delivery. The delivery of naked plasmid DNA (pDNA) is made difficult by the size, hydrophilicity and anionic charge of pDNA molecules, all of which reduce its uptake through the cellular membrane. In addition, pDNA is unstable *in vivo*, owing to its rapid degradation (Scholz & Wagner, 2012). Therefore, many *in vivo* transfection methods such as viral vectors and cationic liposomes have been introduced recently (Pathak et al., 2009; Giacca & Zacchigna, 2012). In spite of their high efficiency, these approaches have some unfavorable properties such as cytotoxicity and immunogenicity (Kawakami et al., 2008). Most carriers are engulfed via endocytosis into the cells. Because pDNA can be depredated in lysosomes, escaping or modulating this pathway is important for efficient gene transfection (Kawabata et al., 1995).

Ultrasound contrast agents (UCAs) such as microbubbles (MBs) and nanobubbles (NBs) have been reported to be effective at enhancing the delivery of pDNA directly to the cytosol via sonoporation (Suzuki et al., 2011; Sirsi & Borden, 2012). Sonoporation is a process that uses UCAs in combination with ultrasound (US) irradiation to create temporary pores in the cellular membrane (Cavalli et al., 2012; Cavalli et al., 2013; Lentacker et al., 2014). This process facilitates the direct delivery of pDNA to the cytosol without endocytosis (Suzuki & Maruyama, 2010; De Temmerman et al., 2011; Un et al., 2011). Moreover, UCAs have unique properties that can be used as advanced carriers in drug delivery. A biocompatible shell made of phospholipids, proteins, or polymers can be loaded with low molecular weight drugs or pDNA (Sirsi & Borden, 2014). The gas core makes it easy to detect the carrier with contrast mode US imaging and also makes it possible to trigger the cavitation needed for sonoporation, leading to site-specific activation (Qin et al., 2009; Unga & Hashida, 2014). NBs are advantageous over MBs due to their smaller size, which allows for better distribution in the blood. NBs can cross through the tiny vasculature that has diameters less than 1 μm (Negishi et al., 2010; Cai et al., 2015; Xie et al., 2015). In addition, due to their small size, NBs can reduce the chances of clinical complications such as vessel blockage and embolisms (Barak & Katz, 2005). As far as the preparation methods of NBs are concerned, most of the previous methods have utilized high sonication power to prepare NBs (Cavalli et al., 2016). A drawback of this method is that NBs produced by sonication have a larger particle-size distribution, with fractions of large bubbles that can cause problems when administered *in vivo* and can increase the chances of embolisms (Stride & Edirisinghe, 2008). Moreover, *in vivo* gene transfection is reliant on the stability of NBs (Alter et al., 2009). Therefore, it is imperative to develop an alternative method that produces stable and uniform NBs, for safe and efficient *in vivo* gene transfection. Based on this context, we developed stable NBs using the mechanical agitation method for efficient *in vivo* gene transfection.

Mechanical agitation can improve the interface between the liquid phase that contains surfactants and the gas phase during the preparation of MBs or NBs. We have previously reported that MBs loaded with doxorubicin can only be stably prepared with the mechanical agitation method (Abdalkader et al., 2015). During the mechanical agitation method, no significant temperature elevation is generated, which is useful for thermo-sensitive materials. Based on this, we hypothesized that by using the mechanical agitation method, stable NBs can be obtained and used for gene transfection applications. In this study, we optimized the preparation conditions of NBs including the agitation time, gas type and lipid concentration. Then, we evaluated the stability of NBs at atmospheric pressures. Finally, the preservation conditions were evaluated, which is important for the efficient sonoporation of pDNA and stability of NBs, both *in vitro* and *in vivo*.

## Material and methods

### Cells and pDNA

Murine colon adenocarcinoma cell line (C26) was obtained from the American Type Culture Collection (ATCC, Manassas, VA). Cells were cultured in Dulbecco’s modified eagle medium obtained from Nissui Pharmaceutical Co., Ltd., (Tokyo, Japan) supplemented with 10% fetal bovine serum and 100 U/ml penicillin/streptomycin at 37 °C in 5% CO_2_. The pCMV-Luc was extracted using an Endofree Plasmid Giga kit (QIAGEN GmbH, Hilden, Germany) as described previously (Un et al., 2010).

### Animals

Female six-week-old ICR mice were purchased from the Shizuoka Agricultural Cooperation Association for Laboratory Animals (Shizuoka, Japan). All experiments were approved by the Animal Experimentation Committee of the Graduate School of Pharmaceutical Sciences, Kyoto University.

### The preparation of NBs

1,2 distearoyl-sn-glycero-3-phosphocholine (DSPC) (Avanti Polar Lipid Inc; Alabaster, AL) and 1,2 distearoyl-sn-glycero-3-phosphoethanolamine-*N*-[amino-(polyethylene glycol)-2000] (PEG_2000_-DSPE) (NOF Co; Tokyo, Japan) with 94:6 molar ratio were dissolved in chloroform, followed by evaporation of the chloroform in a rotary evaporator at 25 °C for 30 min; this was followed by further drying under vacuum at room temperature overnight. The lipid film was hydrated with phosphate buffered saline (PBS) solution at 65 °C for 60 min under mild agitation. The final lipid concentration after hydration was adjusted to 8 mg/ml. The sample was then exposed to bath sonication apparatus for 10 min followed by tip sonication apparatus for 3 min. For preparing NBs, 0.125 ml of liposomes was added to 1.85 ml of PBS in a sterilized vial to obtain a final lipid concentration of 0.5 mg/ml. The air in the vial was removed via a syringe and after capping, 12 ml of nitrogen (GL science Inc; Tokyo, Japan), perfluorobutane (SynQuest Laboratories Inc; Alachua, FL), or perfluoropropane (Takachiho Chemical Industries Co., Tokyo, Japan) was injected. The pressure inside the vial was estimated to be (∼2 atm) using the general law of gases:

(1) PV=nRT

Where *P* is the pressure, *V* is the volume, n is the number of moles, *R* is the universal gas constant and *T* is the temperature in Kelvin. At a fixed temperature, *R* and *T* were considered constant and the pressure was then calculated through the change of gas volume:

(2) $$P_{\mathrm{start}}\times V_{\mathrm{start}}=P_{\mathrm{end}}\times V_{\mathrm{end}}$$

To obtain the NBs, the vials were fixed in a a shaking machine (Ultramate 2, Victoria, Australia) in mixing frequency of 4600 oscillation per min was used for 60 s.

### Characteristics of NBs

The particle size and zeta potential of the liposomes and NBs were determined using a Zetasizer Nano ZS instrument (Malvern Instruments Ltd., Worcestershire, UK). For the optical imaging, diluted NBs were mounted on a 35 mm glass covered dish and images were obtained using the optical filter. The surface area of NBs was calculated from the following equation:

(3) $$\mathrm{SA}\;(\mathrm{bubble})\;=4r^{2}$$

Where SA is the surface area of one NB and *r* is the NB’s radius. Numbers of lipids molecules per one bubble were calculated by the following equation:

(4) $$N=\;\frac{\mathrm{SA}\;(\mathrm{bubble})}{\mathrm{SA}\;(\mathrm{lipid})}$$

Where *N* is number of lipids molecules per one bubble and SA (lipid) is the lipid surface area. In the case of several lipid compositions: SA (lipid) = SA (lipid)_1 _×_ _Mol fraction (lipid)_1_+ SA (lipid)_2 _×_ _Mol fraction (lipid)_2_+ SA (lipid)*_n_*× Mol fraction (lipid)*_n_.*

### Gas content of NBs

Perfluoropropane content analysis was performed by GC–MS as reported previously (Oda et al., 2015).

### In vitro gene transfection and cell cyotoxicity assay

C26 cells were suspended (1 × 10^4^ cells/500 μl) in RPMI medium supplied with 10% FBS. NBs (15 μg) and pDNA (3 μg) were mixed and added to cells, then irradiated via US for 10 and 20 s (2 MHz; 2.5 W/cm^2^; 50% duty; 10 Hz), respectively. After treatment, cells were incubated at 37 °C for 15 h. Luciferase assays in cells were performed according to the method reported previously (Un et al., 2010). Cell cytotoxicity was assessed by carrying out a WST-1 assay using the WST-1 cell proliferation reagent (Roche Diagnostic Corporation, Indianapolis, IN).

### *In vivo* gene transfection into limb muscles

Mice were intravenously injected with 200 and 400 μl of pre-mixed NBs/pDNA that contained 100 or 200 μg of lipids and 50 μg of pDNA. Immediately after injection, the left limb was irradiated with US using Sonopore 4000 (Nepa Gene CO., Ltd., Chiba, Japan) for 60 s (frequency 1 MHz; intensity 1 W/cm^2^; duty 50%; burst rate 10 Hz). Six hours after the injection, the mice were sacrificed and tissues were collected. Luciferase assays in tissues were performed according to the method reported previously (Un et al., 2010).

### Statistical analysis

All data were analyzed as the mean ± SEM. An unpaired, two-tailed distribution Student’s *t*-test was applied, while multi comparisons with control were analyzed using Dennett’s test. Values of *p* < 0.05 were considered statistically significant.

## Results

### Characteristics of NBs

**Figure 1.**
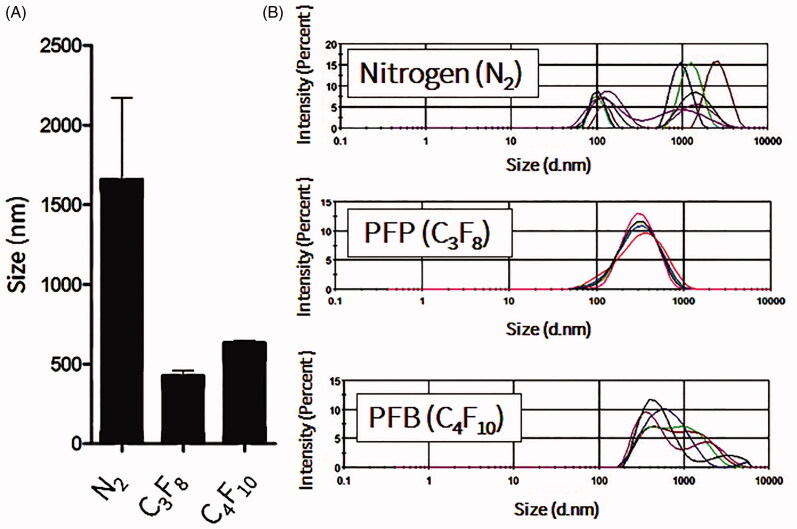
Measurements of nanobubbles (NBs) with different gases. C_3_F_8_ is perfluoropropane gas, C_4_F_10_ is perfluorobutane gas and N_2_ is nitrogen gas. (A) Mean bubble size (*n* = 3; mean ± SEM). (B) Size distribution histograms.

**Figure 2.**
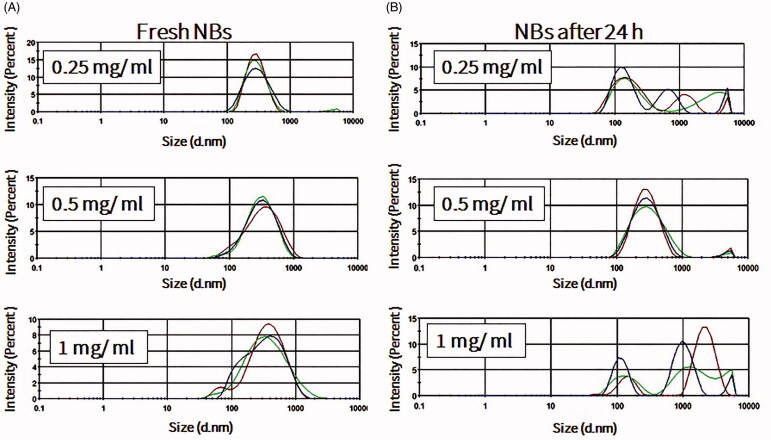
The effects of lipid concentrations on nanobubble (NB) size, distribution and stability at room temperature. (A) Size of freshly prepared NBs (*n* = 3). (B) NBs after storage for 24 h at room temperature (*n *=3).

NBs formed more stably, with uniform size distribution, in the presence of perfluorocarbon gases such as perfluoropropane and perfluorobutane (Figure 1). Mechanical agitation using a shaking machine was applied for 60 s. During agitation, a slight temperature elevation was recorded in the vials, from 22.2 ± 1.5 °C before agitation to 30.1 ± 2.7 °C after agitation. In the presence of oxygen or nitrogen gas, NBs rarely formed and quickly dissolved and disappeared if they did (data not shown). The effect of the lipid concentration on the formation and stability of NBs, at room temperature, was also considered. Freshly prepared NBs with different lipid concentrations had relatively uniform sizes, with an increase in size when the phospholipid concentration increased from 0.2 to 0.5 mg/ml. This observation is consistent with the increase of the NB surface area and number of phospholipids molecules in each bubble. Further increase in the phospholipid concentration to 1 mg/ml did not induce any change in the size of NBs (Supplement 1). However, after 24 h, only NBs that were prepared with a final phospholipid concentration of 0.5 mg/ml had a narrow and uniform size. On the other hand, NBs that were prepared with final phospholipid concentrations of 0.25 and 1 mg/ml were almost completely degraded (Figure 2). The amount of perfluoropropane immediately after uncapping the vial was quantified and estimated to be 54.6 ± 8.4 μl/mg of lipid (*n* = 3).

**Figure 3.**
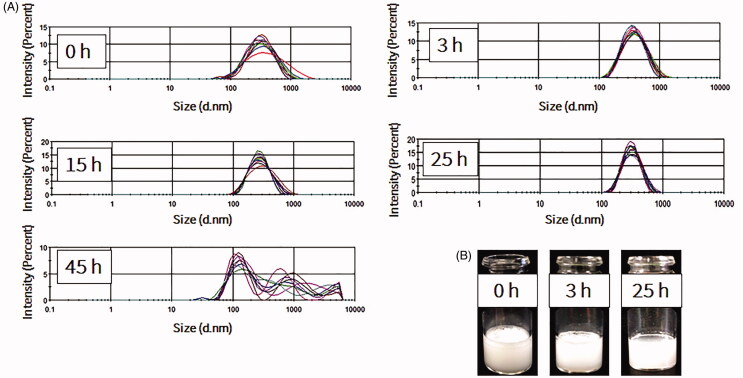
Measurements of nanobubbles (NBs) made with perfluoropropane gas (C_3_F_8_) and left at atmospheric pressure for different time points. (A) NB size distribution histograms; 10 measurements over 25 h. (B) The visual appearance of NBs at 0, 3 and 25 h post exposure of the NBs to the atmospheric pressure conditions.

**Figure 4.**
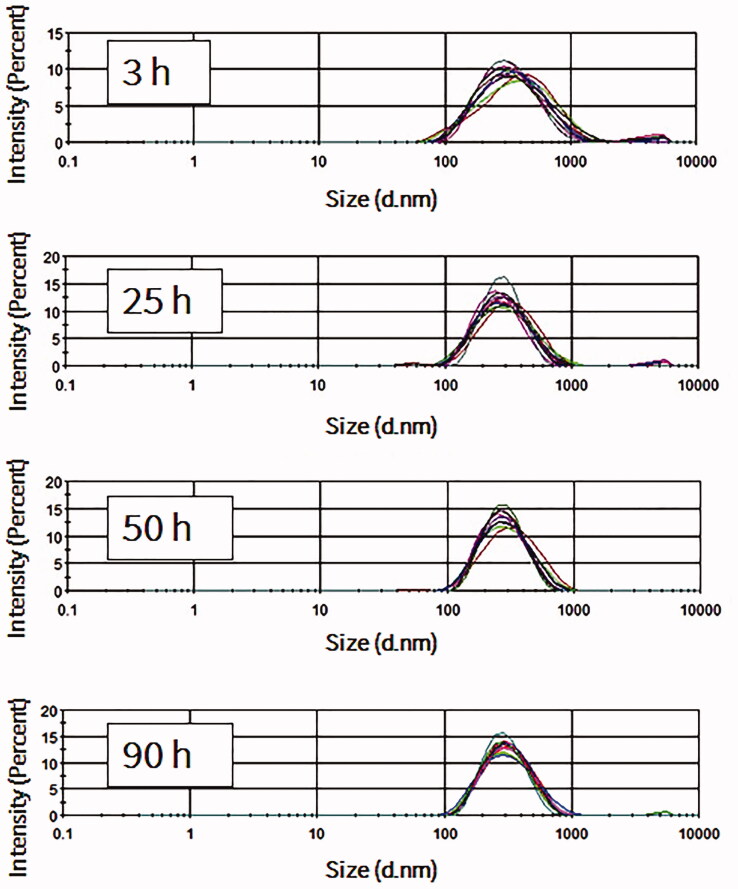
Measurements of nanobubbles (NBs) made with perfluoropropane gas (C_3_F_8_) and left at 4 °C for different time points (*n* = 10).

**Figure 5.**
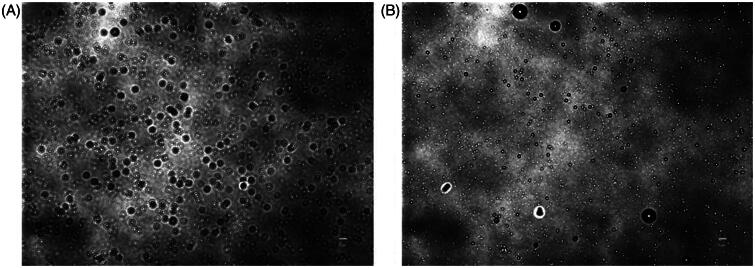
Optical images of nanobubbles (NBs). (A) Freshly prepared NBs. (B) NBs that were kept at 4 °C for 90 h. (scale bar, 5 μm).

The stability of NBs at 4 °C in atmospheric pressure was further investigated over prolonged time durations. For the measurement of size, time 0 was considered as the time immediately after vials were uncapped. NBs had uniform and stable size at room temperature even after 24 h of exposure to atmospheric pressure (*n* = 10; for each time point), with some slight reductions in the size and the correspondent NB surface area (Figure 3). The optical images showed that NBs had a spherical shaped appearance and a diameter of less than 1 μm (Figure 5A). The appearances did not change significantly with time and at 15 h, NBs were spherical in shape at a submicron size. NBs at room temperature could not be preserved more than 24 h, as the bubbles tended to disappear and different size fractions emerged. However, NBs that were kept at 4 °C tended to have a better size distribution. There was some decrease in size after 3 h but this was then sustained for almost 90 h at 4 °C (Figure 4). NBs still existed and the size peaks were unchanged at 90 h. The optical images of preserved NBs at 4 °C also supported these findings and NBs were still characterized with a spherical morphology and a submicron size even at 90 h (Figure 5B).

### The evaluation of NBs ability in enhancing gene transfection *in vitro* and *in vivo*

#### *In vitro* gene transfection and WST-1 assay

**Figure 6.**
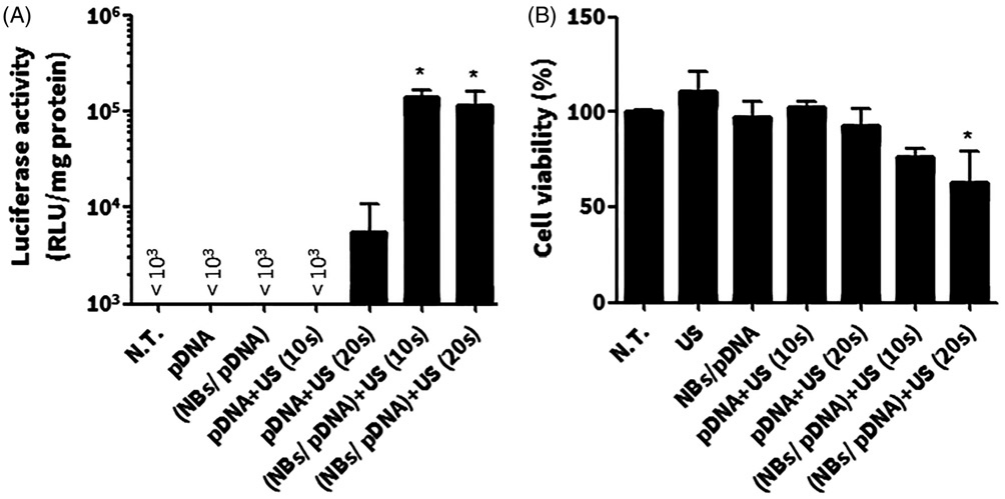
*In vitro* gene expression and cellular damage in C26 cells after various treatments with pDNA. (A) *In vitro* gene transfection. (B) WST-1 assay. Colon C26 cells were treated with nanobubbles (NBs) with pDNA and ultrasound (US) irradiation. Fifteen hours after transfection, cells were harvested and the level of luciferase was evaluated in addition to a WST-1 assay. Each bar represents the mean ± SEM of 3–5 experiments. *p < 0.05 versus the corresponding group of no treatment (NT).

The effect of NBs on the level of *in vitro* gene expression was tested in C26 cells. The result showed that both NBs/pDNA/US10s and NBs/pDNA/US20s significantly enhanced gene expression (*p* < 0.05). In contrast, pDNA only, pDNA/NBs, pDNA/US10s and pDNA/US20s did not induce any significant increases in the level of gene transfection (Figure 6A). The WST-1 assay indicated no significant reductions in cell viability with US, pDNA only, pDNA/NBs and pDNA/NBs/US10s. However, a significant decrease in viable cells was noticed in cells treated with NBs/pDNA/US20s (*p *< 0.05) (Figure 6B).

#### *In vivo* gene expression in limb muscles

**Figure 7.**
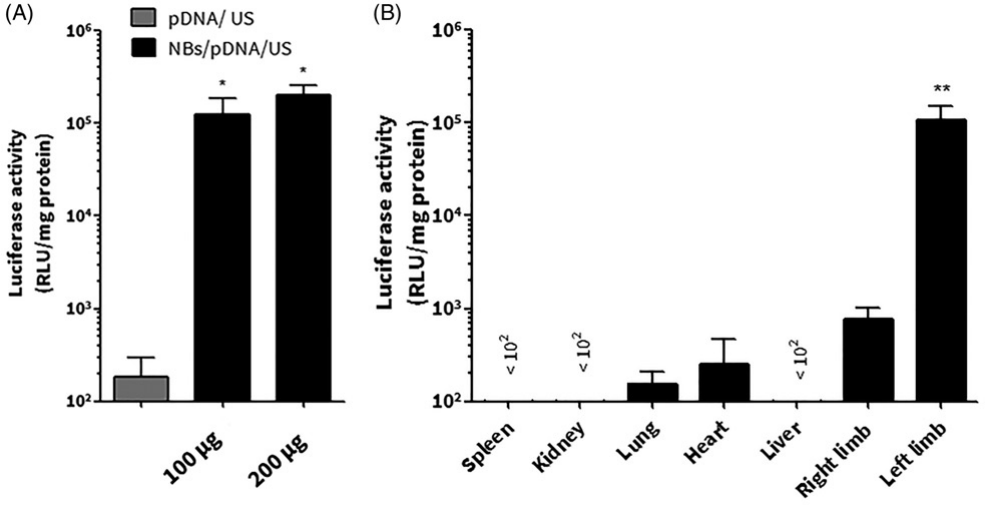
*In vivo* gene transfection in mice limb muscles. (A) The effect of the dose of the nanobubble (NB) on enhancing the gene expression. (B) Tissue selective gene expression. After the intravenous injection of NBs/pDNA, the left muscle was irradiated via ultrasound (US). Each bar represents the mean ± SEM of 3–6 experiments. *p < 0.05 versus the corresponding group of pDNA/US, **p < 0.0001 versus all tissues.

For *in vivo* gene expression, mice left limb muscles were selected as the US irradiation site. NBs were used in two doses: 100 μg lipid/200 μl and 200 μg lipid/400 μl. NBs were capable at inducing significant gene transfection at both doses, while mice treated with pDNA and US had no significant enhancement of gene transfection. For the following experiments, NBs consisted of 200 μg of lipids was employed, pre-mixed with pDNA and intravenously injected (Figure 7A).

Gene transfection in different organs was investigated after the intravenous administration of NBs/pDNA. The left limb muscles that were irradiated with US had the highest level of gene expression (*p* < 0.0001; left limb versus different tissues). In contrast, other tissues that have not been irradiated with US had low level of luciferase activity (Figure 7B).

**Figure 8.**
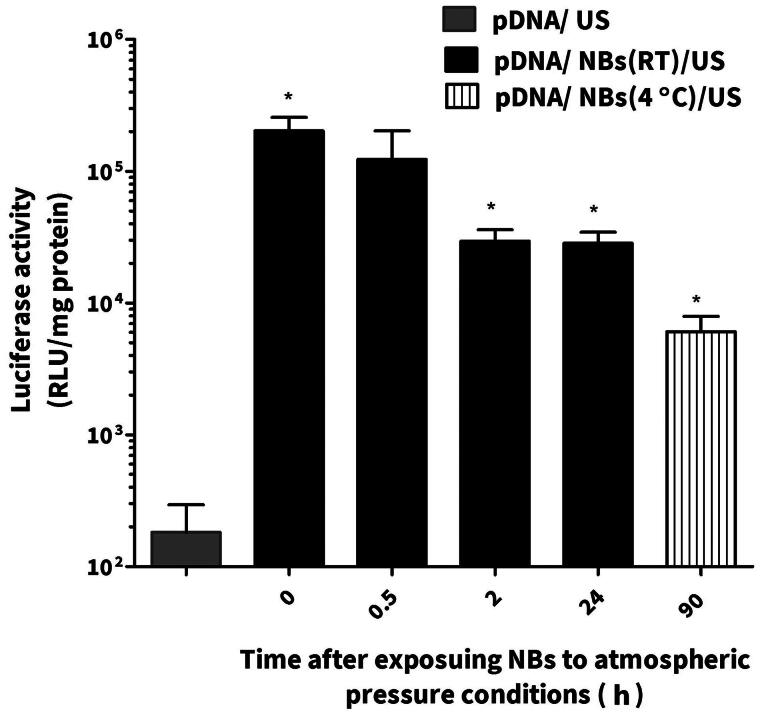
The effect of nanobubble (NB) stability on the *in vivo* gene expression. Each bar represents the mean ± SEM of 3–5 experiments. *p < 0.05 versus the corresponding group of pDNA/US.

NBs were left at the room temperature and at specific time points were mixed with pDNA and administrated intravenously into mice followed with US irradiation on the left limb muscles. The group of animals (*n* = 3–6) that were treated with NBs/pDNA/US had significantly higher gene expression compared those treated with only pDNA/US (*n* = 4). After 2 h, gene expression was slightly decreased. However, the level of gene expression was still significantly higher in groups treated for 24 h with NBs/pDNA/US (*p* < 0.05) compared with mice treated with only pDNA/US. We also evaluated NBs that were preserved at 4 °C for 90 h. Although the gene expression was lower than the groups treated with freshly prepared NBs or even with these left for 24 h at room temperature, the gene expression was significantly higher (*p* < 0.05) compared with mice treated with only pDNA/US (Figure 8).

## Discussion

Recently, many studies have been conducted in order to prepare stable and uniform NBs (Attard, 2013; Cai et al., 2015). However, most of these methods have drawbacks, including the instability of generated NBs and the complexity of the fabrication process (Stride & Edirisinghe, 2008). In this study, we showed that mild mechanical agitation for 60 s was enough to produce NBs with uniform and narrow size distribution. This method did not induce significant elevations in temperature, which implies that this method can be used with materials sensitive to high temperatures or sonication. In order to optimize the best formulation, variables such as the gas type were considered. As shown in Figure 1, NBs had better size distribution and higher stability with perfluorocarbon gases rather than with nitrogen gas. That is because perfluorocarbon gases have lower solubility and diffusivity compared to those of other gases (Szíjjártó et al., 2012). We also used DSPC in NBs to improve their stability, as it has been reported that gas diffusion can be reduced in MBs made of rigid phospholipids such as DSPC (Kwan & Borden, 2012). The amount of encapsulated perfluoropropane gas in NBs was comparably higher than previously reported in NBs that were prepared with the same lipid composition but via the sonication method (Oda et al., 2015). Therefore, by using the mechanical agitation method, NBs with sufficient encapsulation of perfluoropropane gas and narrow size distribution can be produced.

In this study, we succeeded in demonstrating that the lipid concentration has an important impact on the formation and stability of NBs. The increase in the final lipid concentration enhanced the monolayer packing in NBs by incorporating more surfactant in the NBs shell, therefore the number of lipids molecules per NBs can be increased. Sufficient amounts of the surfactant guarantee a drop in surface tension and thus more stable NBs. Less phospholipid (0.25 mg/ml) in the NB shells affected the stability of NBs as they degraded faster. However, more than 1 mg/ml of phospholipid also tended to destabilize NBs, possibly due the over packing in the shells or single micelle formations that might adhere to the NBs. This observation is consistent with a previous report wherein the phospholipid concentration tended to affect the size and characteristics of NBs (Shih & Lee, 2016). Garg et al. (2013) also showed that the phospholipid monolayer in shells of MBs was packed more tightly, leading to the increase of van der Waals interactions between phospholipids molecules. Consequently, there was less gas leakage and MBs were more stable. Therefore, these reports corroborate our results, indicating that the initial phospholipid concentration is important for the optimal stability of NB formulations.

Aged NBs that were left at a temperature of 4 °C were more stable than those left at room temperature. It has been reported that a temperature of 4 °C can increase shell elasticity due to a decrease in activation energy which usually causes shell rupture (Rovers et al., 2016). Despite the fact that this previous report was conducted with MBs that were made with protein shells not phospholipid shells, it is still possible to consider that phospholipid shells may experience a similar phenomenon. Therefore, these findings suggest that changes in the preservation temperature can affect the stability of NBs.

It has been reported that sonoporation can enhance the gene transfection activity of NBs. The process includes the perturbation of the cellular membrane, leading to the direct internalization of pDNA into the cytosol (Karshafian et al., 2009). The complex between bubbles and pDNA is mostly advantageous when NBs are modified with ligands for the purpose of selective targeting or for protecting pDNA degradation *in* the *vivo* conditions. Here, loading pDNA into bubbles is essential. In our study, we aimed to evaluate the caviation effects of NBs and the best way was to simply pre-mix NBs with pDNA before treatment. Our approaches is already based on the previous reports that supported the pre-mix protocol of NBs and pDNA as an effective method for sonoporation mediated gene delivery (Suzuki et al., 2008,2011). To elucidate this effect, *in vitro* gene transfection was first evaluated in C26 cells in order to confirm the transfection ability of NBs. Ten seconds of US irradiation in combination with NBs/pDNA sufficiently enhanced gene expression (Figure 6A) and no significant cytotoxicity was induced (Figure 6B). These results lead us to conclude that the NBs prepared by mechanical agitation method have the potential to induce sonoporation effects with US irradiation, which thus can enhance gene transfection *in vitro*.

*In vivo* conditions involve many critical factors such as shear stress and gas exchanges between bubbles and dissolved gases in the blood that can affect the efficiency of NBs. Consequently, the sonoporation of pDNA can be affected (Wang et al., 2005). Therefore, we tested both fresh and aged NBs in an *in vivo* experiment in mice. As shown in Figure 7(B), the gene expression was specifically enhanced in the left limb muscle of mice treated with NBs/pDNA/US. While, in others organs, the gene expression level was not significantly changed. These results provide evidence that the NBs prepared by the mechanical agitating method have the potential for site-selective *in vivo* gene transfection. On the other hand, aged NBs that were left at room temperature for different periods were also tested in combination with US irritation to discover their sonoporation capability of enhancing gene transfection. Our results indicated that aged NBs kept at room temperature were still effective at enhancing gene expression, even after 24 h, when combined with US. Furthermore, NBs that left at 4 °C for 90 h were also able to enhance gene transfection. Therefore, we believe that aged mechanically formed NBs can still be capable of enhancing *in vivo* gene transfection depending on the preservation conditions. It has been reported that *in vivo* gene transfection is mostly localized to the vascular endothelium inside the limb after the treatment with MBs/pDNA/US (Christiansen et al., 2003; Leong-Poi et al., 2007). In our study, similar process is speculated to be occurred due to NBs cavitation effects that will predominantly increase pDNA localization on the level of endothelial cells.

One limitation of this work is that the imaging aspects of NBs prepared by mechanical agitation method as a contrast agent are not presented in this study. In future studies, the NBs should be evaluated as imaging contrast agents for ultrasonography imaging applications as well as their therapeutic function such as inducing sonoporation effects.

## Conclusions

In the present study, we demonstrated that stable NBs can be prepared through mechanical agitation. NBs with perfluoropropane gas were found to be stable with uniform size for 24 h at room temperature and NBs could be preserved for 90 h at 4 °C. Moreover, the *in vitro* and *in vivo* gene expression was significantly enhanced with NBs/pDNA in combination with US irradiation. In addition, aged NBs that were left for 24 h at room temperature or for 90 h at 4 °C were still functional at enhancing the gene expression in mice limb muscles. These results suggest that the mechanical agitation method is a useful alternative for the development of stable NBs that can be used efficiently for *in vivo* gene transfection.

## Supplementary Material

Supplementary_1.jpg

## Notes

**Supplementary material available online**

## References

[CIT0001] Abdalkader R, Kawakami S, Unga J, et al. (2015). Evaluation of the potential of doxorubicin loaded microbubbles as a theranostic modality using a murine tumor model. Acta Biomater 19:112–18 25795624 10.1016/j.actbio.2015.03.014

[CIT0002] Alter J, Sennoga CA, Lopes DM, et al. (2009). Microbubble stability is a major determinant of the efficiency of ultrasound and microbubble mediated in vivo gene transfer. Ultrasound Med Biol 35:976–84 19285783 10.1016/j.ultrasmedbio.2008.12.015

[CIT0003] Attard P. (2013). The stability of nanobubbles. Eur Phys J Special Topics 1–22. doi:10.1140/epjst/e2013-01817-0

[CIT0004] Barak M, Katz Y. (2005). Microbubbles: pathophysiology and clinical implications. Chest 128:2918–32 16236969 10.1378/chest.128.4.2918

[CIT0005] Cai WB, Yang HL, Zhang J, et al. (2015). The optimized fabrication of nanobubbles as ultrasound contrast agents for tumor imaging. Scientific Rep 5:13725 10.1038/srep13725 PMC4558543 26333917

[CIT0006] Cavalli R, Bisazza A, Trotta M, et al. (2012). New chitosan nanobubbles for ultrasound-mediated gene delivery: preparation and in vitro characterization. Int J Nanomed 7:3309–18 10.2147/IJN.S30912 PMC3396386 22802689

[CIT0007] Cavalli R, Bisazza A, Lembo D. (2013). Micro- and nanobubbles: a versatile non-viral platform for gene delivery. Int J Pharm 456:437–45 24008081 10.1016/j.ijpharm.2013.08.041

[CIT0008] Cavalli R, Soster M, Argenziano M. (2016). Nanobubbles: a promising efficient tool for therapeutic delivery. Therapeut Deliv 7:117–38 10.4155/tde.15.92 26769397

[CIT0009] Christiansen JP, French BA, Klibanov AL, et al. (2003). Targeted tissue transfection with ultrasound destruction of plasmid-bearing cationic microbubbles. Ultrasound Med Biol 29:1759–67 14698343 10.1016/s0301-5629(03)00976-1

[CIT0040] Garg S, Thomas AA, Borden MA. (2013). The effect of lipid monolayer in-plane rigidity on invivo microbubble circulation persistence. Biomaterials 34:6862–70 23787108 10.1016/j.biomaterials.2013.05.053 PMC3762333

[CIT0010] Giacca M, Zacchigna S. (2012). Virus-mediated gene delivery for human gene therapy. J Control Release 161:377–88 22516095 10.1016/j.jconrel.2012.04.008

[CIT0011] Karshafian R, Bevan PD, Williams R, et al. (2009). Sonoporation by ultrasound-activated microbubble contrast agents: effect of acoustic exposure parameters on cell membrane permeability and cell viability. Ultrasound Med Biol 35:847–60 19110370 10.1016/j.ultrasmedbio.2008.10.013

[CIT0012] Kawabata K, Takakura Y, Hashida M. (1995). The fate of plasmid DNA after intravenous injection in mice: involvement of scavenger receptors in its hepatic uptake. Pharm Res 12:825–30 7667185 10.1023/a:1016248701505

[CIT0013] Kawakami S, Higuchi Y, Hashida M. (2008). Nonviral approaches for targeted delivery of plasmid DNA and oligonucleotide. J Pharm Sci 97:726–45 17823947 10.1002/jps.21024

[CIT0014] Kwan JJ, Borden MA. (2012). Lipid monolayer collapse and microbubble stability. Adv Colloid Interf Sci 183–184:82–99 10.1016/j.cis.2012.08.005 22959721

[CIT0015] Lentacker I, De Cock I, Deckers R, et al. (2014). Understanding ultrasound induced sonoporation: definitions and underlying mechanisms. Adv Drug Deliv Rev 72:49–64 24270006 10.1016/j.addr.2013.11.008

[CIT0016] Leong-Poi H, Kuliszewski MA, Lekas M, et al. (2007). Therapeutic arteriogenesis by ultrasound-mediated VEGF165 plasmid gene delivery to chronically ischemic skeletal muscle. Circulat Res 101:295–303 17585071 10.1161/CIRCRESAHA.107.148676

[CIT0017] Negishi Y, Omata D, Iijima H, et al. (2010). Enhanced laminin-derived peptide AG73-mediated liposomal gene transfer by bubble liposomes and ultrasound. Mol Pharm 7:217–26 20020739 10.1021/mp900214s

[CIT0018] Oda Y, Suzuki R, Mori T, et al. (2015). Development of fluorous lipid-based nanobubbles for efficiently containing perfluoropropane. Int J Pharm 487:64–71 25841568 10.1016/j.ijpharm.2015.03.073

[CIT0019] Pathak A, Patnaik S, Gupta KC. (2009). Recent trends in non-viral vector-mediated gene delivery. Biotechnol J 4:1559–72 19844918 10.1002/biot.200900161

[CIT0020] Qin S, Caskey CF, Ferrara KW. (2009). Ultrasound contrast microbubbles in imaging and therapy: physical principles and engineering. Phys Med Biol 54:R27–57 19229096 10.1088/0031-9155/54/6/R01 PMC2818980

[CIT0021] Rovers TAM, Sala G, van der Linden E, Meinders MBJ. (2016). Effect of temperature and pressure on the stability of protein microbubbles. ACS Appl Mater Interfaces 8:333–40 26619225 10.1021/acsami.5b08527

[CIT0022] Scholz C, Wagner E. (2012). Therapeutic plasmid DNA versus siRNA delivery: common and different tasks for synthetic carriers. J Control Release 161:554–65 22123560 10.1016/j.jconrel.2011.11.014

[CIT0023] Shih R, Lee AP. (2016). Post-formation shrinkage and stabilization of microfluidic bubbles in lipid solution. Langmuir 32:1939–46 26820229 10.1021/acs.langmuir.5b03948

[CIT0024] Sirsi SR, Borden MA. (2012). Advances in ultrasound mediated gene therapy using microbubble contrast agents. Theranostics 2:1208–22 23382777 10.7150/thno.4306 PMC3563148

[CIT0025] Sirsi SR, Borden MA. (2014). State-of-the-art materials for ultrasound-triggered drug delivery. Adv Drug Deliv Rev 72:3–14 24389162 10.1016/j.addr.2013.12.010 PMC4041842

[CIT0026] Stride E, Edirisinghe M. (2008). Novel microbubble preparation technologies. Soft Matter 4:2350–9

[CIT0027] Suzuki R, Oda Y, Utoguchi N, Maruyama K. (2011). Progress in the development of ultrasound-mediated gene delivery systems utilizing nano- and microbubbles. J Control Release 149:36–41 20470839 10.1016/j.jconrel.2010.05.009

[CIT0028] Suzuki R, Takizawa T, Negishi Y, et al. (2008). Tumor specific ultrasound enhanced gene transfer in vivo with novel liposomal bubbles. J Control Release 125:137–44 18035442 10.1016/j.jconrel.2007.08.025

[CIT0029] Suzuki R, Maruyama K. (2010). Effective in vitro and in vivo gene delivery by the combination of liposomal bubbles (bubble liposomes) and ultrasound exposure. Methods Mol Biol (Clifton, NJ) 605:473–86 10.1007/978-1-60327-360-2_33 20072902

[CIT0030] Szíjjártó C, Rossi S, Waton G, Krafft MP. (2012). Effects of perfluorocarbon gases on the size and stability characteristics of phospholipid-coated microbubbles: Osmotic effect versus interfacial film stabilization. Langmuir 28:1182–9 22176688 10.1021/la2043944

[CIT0031] De Temmerman ML, Dewitte H, Vandenbroucke RE, et al. (2011). MRNA-lipoplex loaded microbubble contrast agents for ultrasound-assisted transfection of dendritic cells. Biomaterials 32:9128–35 21868088 10.1016/j.biomaterials.2011.08.024

[CIT0032] Un K, Kawakami S, Suzuki R, et al. (2010). Development of an ultrasound-responsive and mannose-modified gene carrier for DNA vaccine therapy. Biomaterials 31:7813–26 20656348 10.1016/j.biomaterials.2010.06.058

[CIT0033] Un K, Kawakami S, Yoshida M, et al. (2011). The elucidation of gene transferring mechanism by ultrasound-responsive unmodified and mannose-modified lipoplexes. Biomaterials 32:4659–69 21481454 10.1016/j.biomaterials.2011.03.013

[CIT0034] Unga J, Hashida M. (2014). Ultrasound induced cancer immunotherapy. Adv Drug Deliv Rev 72:144–53 24680708 10.1016/j.addr.2014.03.004

[CIT0035] Wang X, Liang HD, Dong B, et al. (2005). Gene transfer with microbubble ultrasound and plasmid DNA into skeletal muscle of mice: comparison between commercially available microbubble contrast agents. Radiology 237:224–9 16081853 10.1148/radiol.2371040805

[CIT0036] Xie X, Lin W, Liu H, et al. (2015). Ultrasound-responsive nanobubbles contained with peptide-camptothecin conjugates for targeted drug delivery. Drug Deliv 7544:1–9 10.3109/10717544.2015.1077289 26289216

