# Supplementary figures and images for: The development of mechanically formed stable nanobubbles intended for sonoporation-mediated gene transfection
Source: Drug Deliv. 2017 Feb 6;24(1):320–7. doi: 10.1080/10717544.2016.1250139 (PMC8241156; doi:10.1080/10717544.2016.1250139)

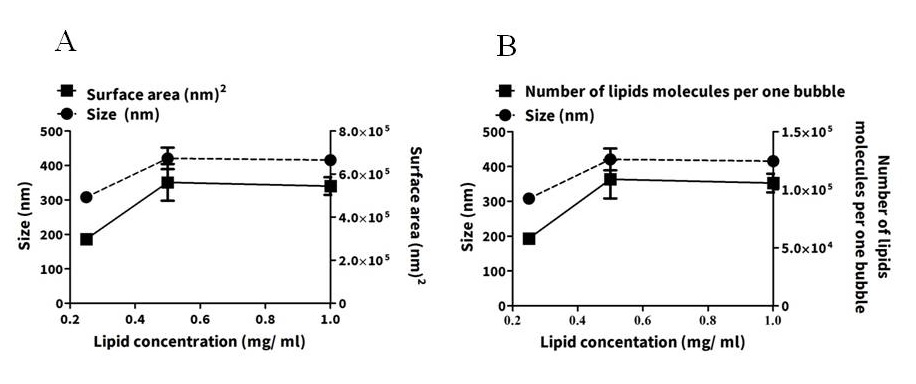

Supplement: Supplementary_1.jpg [file IDRD_A_1250139_SM5849.jpg]
